# WALINET: A water and lipid identification convolutional neural network for nuisance signal removal in MR spectroscopic imaging

**DOI:** 10.1002/mrm.30402

**Published:** 2024-12-31

**Authors:** Paul J. Weiser, Georg Langs, Stanislav Motyka, Wolfgang Bogner, Sébastien Courvoisier, Malte Hoffmann, Antoine Klauser, Ovidiu C. Andronesi

**Affiliations:** ^1^ Athinoula A. Martinos Center for Biomedical Imaging Massachusetts General Hospital Boston Massachusetts USA; ^2^ Department of Radiology Massachusetts General Hospital, Harvard Medical School Boston Massachusetts USA; ^3^ Computational Imaging Research Lab–Department of Biomedical Imaging and Image‐guided Therapy Medical University of Vienna Vienna Austria; ^4^ High Field MR Center–Department of Biomedical Imaging and Image‐Guided Therapy Medical University of Vienna Vienna Austria; ^5^ Center for Biomedical Imaging (CIBM) Geneva Switzerland; ^6^ Department of Radiology and Medical Informatics, University of Geneva Geneva Switzerland; ^7^ Advanced Clinical Imaging Technology Siemens Healthineers International AG Lausanne Switzerland

**Keywords:** brain, metabolite quantification, mr spectroscopic imaging, ultrahigh‐field mr, water and lipid removal

## Abstract

**Purpose:**

Proton magnetic resonance spectroscopic imaging (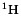
‐MRSI) provides noninvasive spectral‐spatial mapping of metabolism. However, long‐standing problems in whole‐brain 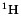
‐MRSI are spectral overlap of metabolite peaks with large lipid signal from scalp, and overwhelming water signal that distorts spectra. Fast and effective methods are needed for high‐resolution 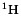
‐MRSI to accurately remove lipid and water signals while preserving the metabolite signal. The potential of supervised neural networks for this task remains unexplored, despite their success for other MRSI processing.

**Methods:**

We introduce a deep learning method based on a modified Y‐NET network for water and lipid removal in whole‐brain 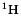
‐MRSI. The WALINET (WAter and LIpid neural NETwork) was compared with conventional methods such as the state‐of‐the‐art lipid L2 regularization and Hankel–Lanczos singular value decomposition (HLSVD) water suppression. Methods were evaluated on simulated models and in vivo whole‐brain MRSI using NMRSE, SNR, CRLB, and FWHM metrics.

**Results:**

WALINET is significantly faster and needs 8s for high‐resolution whole‐brain MRSI, compared with 42min for conventional HLSVD+L2. WALINET suppresses lipid and water in the brain by 25–45 and 34–53‐fold, respectively. WALINET has better performance than HLSVD+L2, providing: (1) more lipid removal with 41% lower NRMSE; (2) better metabolite signal preservation with 71% lower NRMSE in simulated data; 155% higher SNR and 50% lower CRLB in in vivo data. Metabolic maps obtained by WALINET in healthy subjects and patients show better gray‐/white‐matter contrast with more visible structural details.

**Conclusions:**

WALINET has superior performance for nuisance signal removal and metabolite quantification on whole‐brain 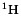
‐MRSI compared with conventional state‐of‐the‐art techniques. This represents a new application of deep learning for MRSI processing, with potential for automated high‐throughput workflow.

## 1 INTRODUCTION

Proton magnetic resonance spectroscopic imaging (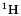
‐MRSI) has great potential as a metabolic imaging technique that can measure the intrinsic metabolite concentrations across the whole‐brain without the need to administer molecular agents.^1^ Proton‐based MR spectroscopy, compared with X‐nuclei spectroscopy, has a significantly higher SNR and is ubiquitously available on all MRI scanners equipped with standard hardware and software. Consequently, it represents the vast majority of MRS performed in clinical studies and research applications. In specific pathology, it allows the detection of metabolic abnormalities in the brain before anatomical lesions are visible.^2^ Therefore, it is valuable for disease investigation such as brain tumor identification, classification, and treatment response assessment.^3^ It further enables the study of neurochemistry alterations and disease mechanisms in neuropsychiatry.^4^ However, the clinical potential of 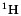
‐MRSI is not fully realized due to complex experimental factors that reduce data quality such as large artifacts from the overwhelming water and lipid signals.^5^, ^6^ Hence, efficient solutions to these technical problems can have great and widespread impact on the use of 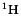
‐MRSI in clinical applications.

Since water is a major component of all brain tissues (70%–85%), the signal strength of water artifacts is particularly large, resulting in amplitudes 3–4 orders of magnitude greater than those of metabolites. Although the water peak at 4.68 ppm does not directly overlap with the peaks of the major metabolites in the aliphatic region (0.9–4.2 ppm), frequency modulation due to timing of the pulse sequence acquisition can lead to large water side‐bands that overlap metabolite peaks and cause significant baseline distortions. Additionally, the water suppression pulses change the shape of the water peak from an absorption symmetric peak shape to an asymmetric peak shape where the tails are larger than the center of peak.

The removal of lipid signal originating from the scalp region presents with even greater challenges.^7^ The lipid spectrum is complicated, having multiple peaks that fully overlap with aliphatic region and are 1–2 orders of magnitude stronger than metabolite peaks. Furthermore, the lipid signal originates from scalp areas with very inhomogeneous $B_{0}$ field, hence they are much broader than water and metabolite peaks from inside the brain.

Methods enabling suppression of water and lipid contamination can be categorized into several groups^7^: techniques leveraging specific RF‐pulse or sequence designs to invert, nullify, or saturate water or lipid resonances^8^, ^9^, ^10^, ^11^, ^12^, ^13^, ^14^, ^15^ approaches utilizing dedicated hardware to spoil the scalp signal,^16^, ^17^ and post‐acquisition methods employing spatial or spectral priors for lipid contamination removal. The latter category in the case of lipids removal can be divided into methods using lipid signal extrapolation,^18^ dual‐density reconstruction,^19^, ^20^ lipid‐basis penalty,^21^, ^22^ subspace reconstruction methods based on specific spatial supports and spectral decomposition,^23^, ^24^, ^25^ while in the case of water removal includes Hankel matrix singular value decomposition,^26^ subspace reconstruction,^23^ Lowner tensorization,^27^ and water‐basis penalty.^28^

In practice, whole‐brain 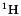
‐MRSI has residual water and lipid signals present even after using special pulse sequences and hardware designed to suppress them, hence requiring post‐acquisition processing methods. Optimized processing methods for nuisance signal removal are particularly relevant for 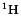
‐MRSI at 7T, because $B_{0}$, $B_{1}+$ inhomogeneity and high SAR at ultrahigh field make lipid and water suppression during acquisition prohibitive and inefficient.

Due to fundamentally different spatial distribution, spectral ranges, and signal shapes of water and lipids, existing postprocessing methods usually only allow the removal of water *or* lipid signals, but not both at the same time.

Recently, closed‐form solutions have been developed for lipid removal.^29^, ^30^ Closed‐form solutions are derived by applying a linear operator on each individual spectrum and are faster compared with iterative lipid suppression algorithms. However, the linear operator method has some drawbacks too: (1) it is subject specific, (2) requires accurate anatomical mask delineation, and (3) may not work if the orthogonal assumption and linear superposition between lipids and metabolites are not met.

Hence, long processing times and the requirement for tedious subject‐specific parameter optimizations such as the L2 regularization factor to balance nuisance signal suppression with the preservation of metabolic signals, make difficult the application of existing nuisance signal removal methods for fast, robust, and automated MRSI processing pipelines.

In recent years, deep learning‐based methods applied to mitigate MRS challenges have gained popularity, enabling robust applications and eliminating the need for complex parameter optimizations. Thereby, applications can be categorized into deep learning‐based artifact removal, denoising, low rank,^31^, ^32^, ^33^ and spectral quantification.^34^, ^35^, ^36^, ^37^, ^38^

Motivated by these developments, we introduce a convolutional neural network for the identification of water & lipid (WALINET: WAter and LIpid neural NETwork) signals in 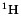
‐MRSI spectra, and evaluated its performance in simulations and in vivo data measured in human participants.

## 2 METHODS

### 2.1 Strategy

The problem can be formulated using two distinct inputs $x_{1}$ and $x_{2}$: (1) the original MRS spectrum ($x_{1}$) containing metabolite signal m contaminated with lipid l and water w signal, and (2) the spectrum ($x_{2}$) subjected to a projection onto the lipid subspace, 1−ℒ, using lipid L2 regularization^29^ approach with $ℒ={\left(1+\beta LL^{H}\right)}^{-1}$. L is a matrix containing the in vivo lipid signal obtained from the scalp mask of each subject and β is the regularization parameter. The derived operator ℒ is an approximation of a projection whose kernel is given by the linear span of L. 1−ℒ, with 1 being an identity matrix, is a projection onto the lipid subspace span(L) (see references for further details). A separate lipid projection operator is calculated for each subject. The network inputs are defined as

(1) $$\begin{array}{ll} x_{1} & =m+l+w, \end{array}$$

(2) $$\begin{array}{ll} x_{2} & =(1-ℒ)x_{1}. \end{array}$$

The decontaminated solution ($\tilde{m}$) is obtained in two steps, first the network 𝒴 predicts the spectrum y, thereby approximating lipid l and water w signal. Subsequently, the spectrum y is subtracted from $x_{1}$, the original water‐ and lipid‐contaminated MRSI spectrum,

(3) $$\begin{array}{ll} 𝒴(x_{1},x_{2}) & =y\approx l+w, \end{array}$$

(4) $$\begin{array}{ll} \tilde{m} & =x_{1}-y. \end{array}$$

A similar strategy is employed for lipid removal only, with the exception that water signal is omitted.

### 2.2 Network architecture

**FIGURE 1:**
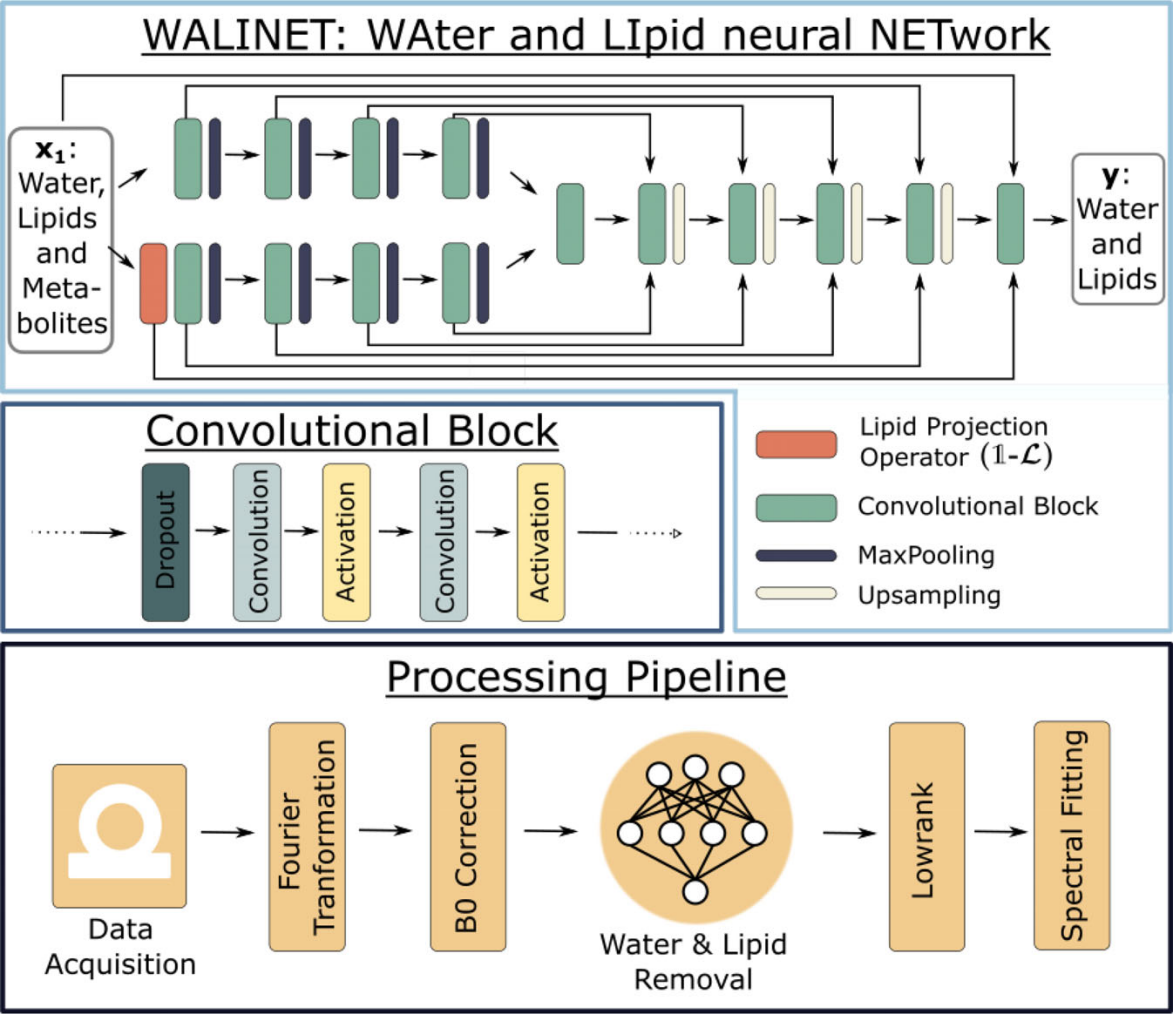
WAter and LIpid neural NETwork (WALINET): Top: Y‐Net architecture containing four convolutional blocks in each encoder and decoder, followed by a MaxPooling or Upsampling layer. Additional convolutional blocks are incorporated in the bottleneck and as a final layer. A lipid projection operator at the beginning of the second encoder enhances the distinguishability between metabolites and lipids. Bottom: The WALINET is embedded into the MRSI processing pipeline shown at the bottom, which includes Fourier transformation, B0 correction, low‐rank model, and spectral quantification.

WALINET employs a Y‐Net convolutional neural network structure,^39^ depicted in Figure 1, and characterized by the use of two encoders instead of one. Note, that the same network architecture can be used and trained only for lipid removal, which we call LIPNET (LIpid neural NETwork).

The Y‐Net architecture enables enhanced contextual understanding by integrating features from different branches, leading to improved understanding of complex structures within the data. The results show an improvement compared with the U‐Net^39^ and enable the encoding of different features in each encoder.^40^

Each encoder (ℰ) and decoder (𝒟) of the Y‐Net (𝒴) comprised four convolutional blocks, each consisting of two convolutional layers, PReLU activation functions,^41^ dropout with a rate of 0.01, and MaxPooling/upsampling with a factor of 2. Skip connections are implemented between the encoders and decoder. An additional convolutional block is incorporated in the bottleneck region and after the decoder. The outputs of the encoders are concatenated and forwarded to the bottleneck convolutional block in the decoder.

(5) $$\begin{array}{ll} 𝒴(x_{1},x_{2}) & =𝒟({ℰ}_{1}(x_{1}),{ℰ}_{2}(x_{2})) \end{array}$$

(6) $$\begin{array}{ll} & =𝒟({ℰ}_{1}(x_{1}),{ℰ}_{2}((1-ℒ)x_{1})). \end{array}$$

The kernel size of each block is set to 7. The number of channels in the first convolutional block is set to 16 and is doubled/halved after every MaxPooling/Upsampling layer.

### 2.3 Training data

The training spectra were generated through a multistep process, where metabolite spectra were simulated and combined with experimentally measured lipid and water spectra from in vivo human data. This approach was chosen because: (1) metabolite spectra can be realistically simulated for an extremely large range of very diverse parameters; (2) the lipid and water spectra are affected by a complex combination of experimental factors that are hard to be fully accounted in simulations, hence more realistic spectra can be extracted from measured data.

Spectra for 25 common 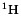 metabolites were simulated using a physical model for the coupled spin systems.^42^, ^43^ An extensive dataset of $1.9\times 10^{6}$ metabolite spectra was simulated for a wide range of concentrations, linewidths, noise levels, and baselines. Water and lipid signals were extracted from 19 subjects, including 2 glioma patients. The MRSI data were acquired with the 3D 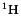
‐FID‐ECCENTRIC sequence described in Section 2.5. $10^{5}$ lipid & water spectra were extracted from each subject, resulting in a total $1.9\times 10^{6}$ lipid & water spectra that were randomly combined with metabolite spectra for training. Additionally, a validation dataset included four other subjects. Further details regarding the generation of the training data are provided in the Supporting Information. A total of 23 subjects were scanned at the Athinoula A. Martinos Center for Biomedical Imaging with informed consent and a protocol approved by the IRB committee of MGH (Protocol 2013P001195).

### 2.4 Training procedure

The training spectra were subjected to further augmentation and normalization before being forwarded to the neural network. Online data augmentation during training was achieved by multiplication with a random phase

(7) $$\begin{array}{l} \phi =e^{i\omega },\;\omega \in [0,2\pi ] \end{array}$$

to each spectrum. Normalization was performed by dividing the input and ground‐truth spectra by an approximation of the energy 𝔼 of the underlying metabolic signal. Therefore, the root‐sum‐squared‐error between the two input spectra was computed.

(8) $$\begin{array}{l} 𝔼=\sqrt{{\left|x_{1}-x_{2}\right|}^{T}\left|x_{1}-x_{2}\right|}. \end{array}$$

After augmentation and normalization, the spectra were separated into real and imaginary part, which were treated as separate channels during training. Mean‐squared error was employed as training loss and computed on the separated real and imaginary channels.

(9) $$\begin{array}{l} \text{Loss}=\text{MSE}\left(𝒴\left(\frac{x_{1}\phi }{E},\frac{x_{2}\phi }{E}\right),\frac{y\phi }{E}\right). \end{array}$$

The network was trained for 400 epochs, using the Adam^44^ optimizer with a learning rate of 0.01, which was quartered every 50 epochs. The exponential decay rates of the first and second momentum of Adam $\beta_{1}$ & $\beta_{2}$ were set to 0.9 and 0.999. The network was implemented using PyTorch 2.0.1 and CUDA 11.7 packages in Python 3.8.13. The model training was performed on a PowerEdge R7525 server (Dell) with 64 CPU cores (AMD EPYC7542 2.90GHz, 128M Cache, DDR4‐3200), 512 GB CPU RAM (RDIMM, 3200MT/s), 3 GPU NVIDIA Ampere A40 (PCIe, 48GB GPU RAM) running Rocky Linux release 8.8 (Green Obsidian).

### 2.5 Data acquisition

In vivo MRSI data were acquired with 2D 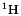
‐FID Cartesian phase encoded^45^ and 3D 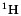
‐FID ECCENTRIC^46^ pulse sequences using a 7T MR scanner (MAGNETOM Terra, Siemens Healthineers, Forchheim, Germany) and a 1Tx/32Rx head coil (NovaMedical, Wilmington, MA, USA).

2D 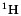
‐FID Cartesian MRSI data were acquired on two subjects with matrix 53×41, 164×212 mm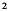 field‐of‐view (FoV), 4×4 mm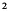 in‐plane voxel size, 10 mm slice thickness, spectral bandwidth of 4 kHz, and 512 FID points.

3D 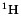
‐FID‐ECCENTRIC^46^ was acquired on 21 subjects with 64×64×31 matrix, 220×220×105 mm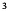 FoV, 3.4×3.4×3.4 mm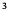 voxel size, spectral bandwidth of 2326 Hz, and 453 FID points.

For both sequences 0.9 ms echo time (TE) and 275 ms repetition‐time (TR) were used, resulting in 18 min:40 s for 3D ECCENTRIC and 7 min:48 s for 2D Cartesian. Further details are provided in the Supporting Information.

### 2.6 Processing pipeline

The reconstruction and processing of 2D and 3D data are performed by a similar pipeline (Figure 1). However, the ECCENTRIC MRSI requires additional steps because of the non‐Cartesian k‐space sampling as further explained in the Supporting Information.

For comparison of WALINET and LIPNET performance, conventional nuisance signal removal was performed with HLSVD for water^26^ and L2 regularization for lipids.^29^ The HLSVD retained the 32 largest eigenvalues of the Hankel matrix and the water removal was applied in the frequency range of 4.7 ± 0.5 ppm. L2 regularization used lipid signals extracted from the skull mask of the subject with the regularization parameter β individually adjusted for each subject to achieve a mean absolute diagonal value of its lipid suppression operator $ℒ={\left(1+\beta LL^{H}\right)}^{-1}$ at an arbitrary value of 0.938,

(10) $$\begin{array}{l} \text{mean}(|\text{diag}(ℒ)|)∼0.938. \end{array}$$

This value was selected as the optimal trade‐off between minimizing metabolite alteration and maximizing lipid suppression.

Following the water and lipid removal, a low‐rank model was employed assuming separable spatial $U_{n}(r)$ and temporal $V_{n}(t)$ components of the metabolite signal,

(11) $$\begin{array}{l} m(r,t)=\overset{K}{\underset{n=1}{\sum }}U_{n}(r)V_{n}(t) \end{array}$$

with K the rank of the model set to 40.

As final step, metabolic quantification was carried out by LCModel^47^ spectral fitting.

## 3 RESULTS

### 3.1 Simulation results

**FIGURE 2:**
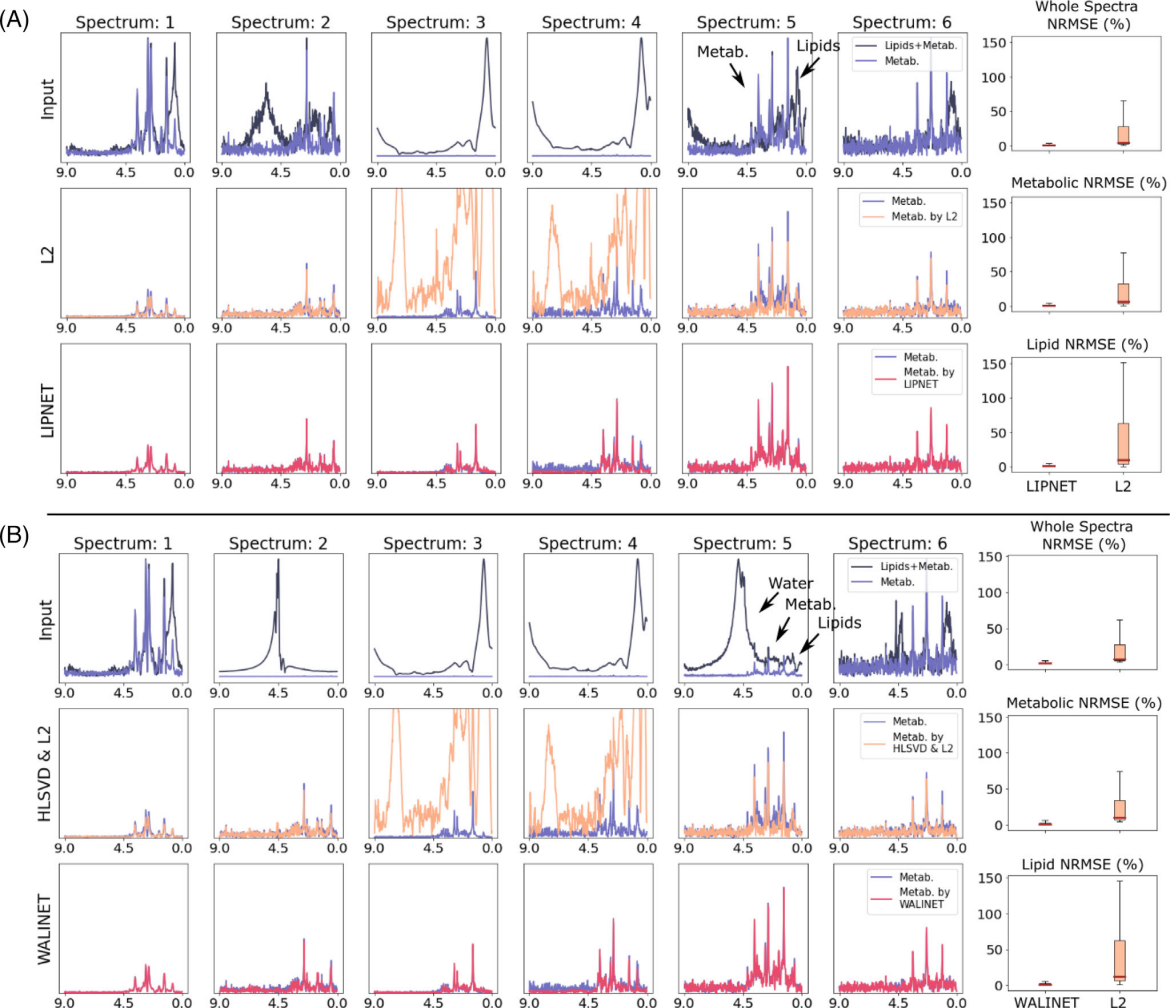
Simulation results. (A) Comparison of lipid suppression by LIPNET and L2. Input spectra contaminated by lipids are shown in the first row (black), second row shows metabolite spectra recovered by L2 regularization (orange), and third row shows the metabolite spectra recovered by LIPNET (red). (B) Input spectra contaminated by water and lipids are shown on the first row (black), in the second row metabolite spectra processed with HLSVD+L2 are displayed, and the metabolite spectra predicted by WALINET (red) are plotted below. The ground‐truth metabolite spectra (blue) are overlaid in all spectral plots. Normalized root‐mean‐squared error (NRMSE) is computed for the whole spectrum (9.0–0.0 ppm), the metabolic range (4.2–1.9 ppm), and the lipid range (1.9–0.7 ppm) for each method LIPNET or L2 in (A) and WALINET or HLSVD+L2 in (B). Separate evaluation of specific spectral ranges allows an individual assessment of the preservation of metabolic signals, as well as the effectiveness of lipid and water suppression. Arrows indicate the position of the main peaks of water, lipids, and metabolites.

In Figure 2, the results of LIPNET and WALINET on simulated data are compared with L2 and HLSVD+L2, respectively. Evaluation data were created by merging 100 000 simulated metabolite spectra with in vivo water & lipid signals (for WALINET) or only lipid signals (for LIPNET) extracted from a subject excluded from the training data. Results on evaluation spectra contaminated only by lipid are shown in Figure 2A, and by water & lipid are shown in Figure 2B. We obtained more agreement between ground‐truth metabolite spectra and the predicted metabolite spectra in the case of LIPNET and WALINET than in the case of L2 and HLSVD+L2 regularization. Spectra obtained by L2 regularization tend to show more residual lipid signal and more suppression of the NAA peak compared with WALINET and LIPNET. Quantitatively, boxplots of the normalized root mean square error (NRMSE) show that WALINET and LIPNET remove more lipid signal (interquartile NRMSE 0.86%–2.69%) while preserving more metabolite signal (interquartile NRMSE 0.62%–1.45%) compared with L2 (lipid interquartile NRMSE 3.68%–6.45% and metabolite interquartile NRMSE 1.04%–4.11%).

### 3.2 In vivo results

In vivo performance was tested on Cartesian‐encoded 2D MRSI and ECCENTRIC‐encoded 3D MRSI data from human volunteers. The speed of all methods for nuisance signal removal was timed on in vivo high‐resolution 3D MRSI data and showed considerably faster times for WALINET compared with HLSVD+L2, as listed in Table 1. The 2D MRSI was used to test the generalizability of WALINET and LIPNET trained on 3D MRSI data.

**TABLE 1:** Processing times of WALINET, water HLSVD, and lipid L2 regularization for 1 CPU core and parallelized for 10 CPU cores.

	WALINET (mm:ss)	HLSVD+L2 (mm:ss)	HLSVD (mm:ss)	L2 (mm:ss)
10 cores	00:08	03:08	03:05	00:03
1 core	00:08	42:49	42:46	00:03

*Note*: LIPNET is based on the same Y‐NET architecture as WALINET and therefore requires the same processing time. Parallelization of L2 and WALINET across several cores is not possible, therefore equivalent processing times are given in each row for these two methods.

**FIGURE 3:**
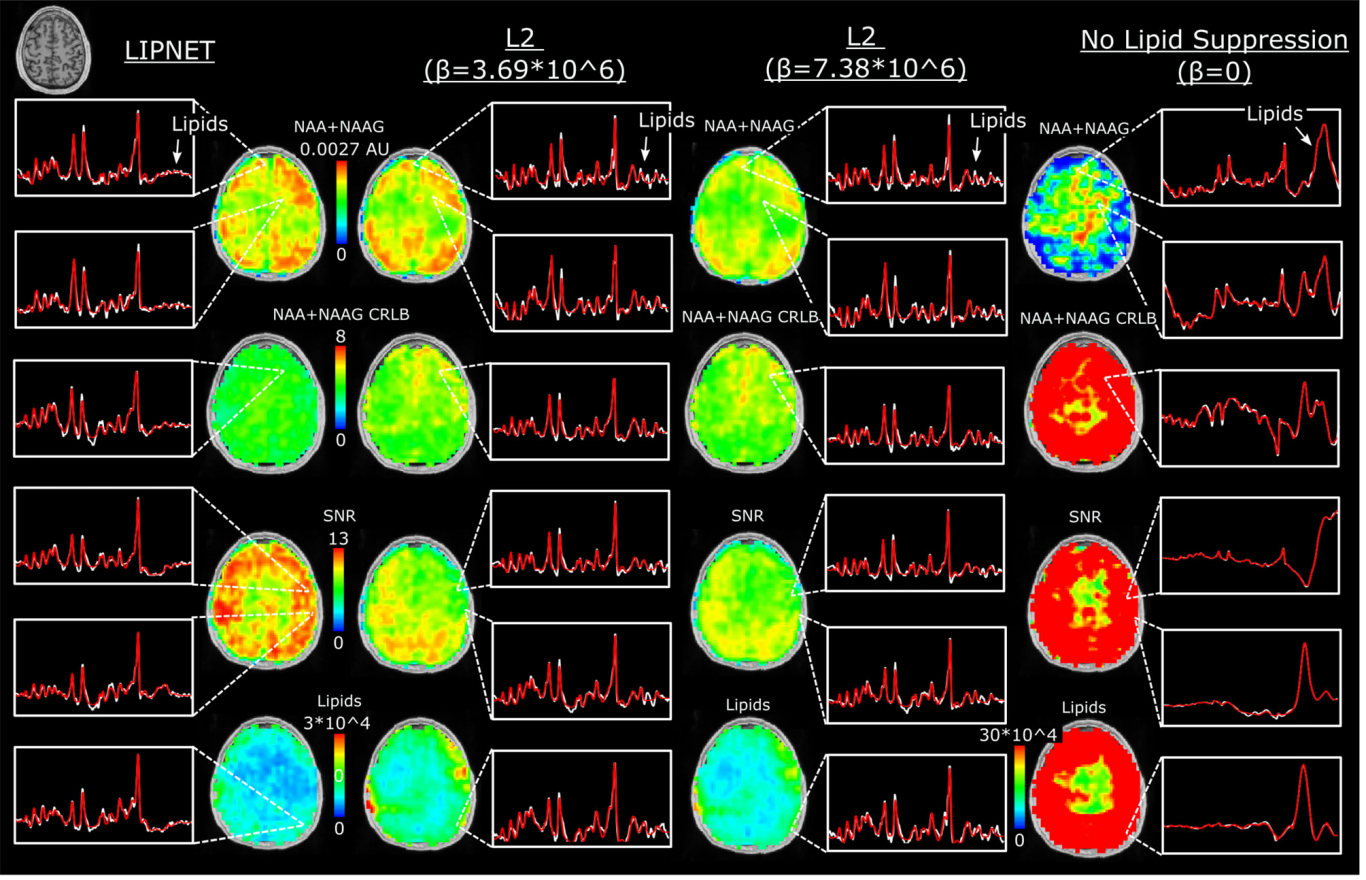
Comparison of lipid removal on in vivo 2D MRSI by LIPNET and L2 for three values of the regularization parameter β, the optimal value ($3.69*10^{6}$), double the optimal value, and zero for no lipid suppression. Metabolic maps are shown for NAA+NAAG, the corresponding CLRB, SNR computed by LCModel,^47^ and residual lipid signal. Spectra from several brain voxels are shown for each method, the white trace shows the measured spectrum, and the red trace shows LCModel fit.

First, we studied the effects of different lipid suppression methods. Figure 3 compares lipid removal by LIPNET and L2 regularization on in vivo 2D MRSI data, while the water removal has been done by HLSVD for all the tests. For L2 two regularization parameters were used: 1) $\beta =3.69*10^{6}$ optimized for the 0.938 mean absolute diagonal value of the lipid suppression operator; and 2) $\beta =7.38*10^{6}$, which doubles lipid regularization parameter for stronger lipid suppression. In addition, results obtained with no lipid suppression (β=0) are presented.

Metabolic maps of NAA+NAAG obtained with lipid suppression methods show similar structural features with good contrast between gray–white matter. However, some differences can be observed: (1) L2 regularization produces lower levels for NAA+NAAG maps compared with NAA+NAAG levels obtained by LIPNET; (2) the metabolic maps obtained with the strongest L2 regularization ($\beta =7.38*10^{6}$) have the lowest metabolite levels. The superior performance of LIPNET is also confirmed by the spectral quality maps, which show 10%–50% smaller CRLB and 155% higher SNR compared with L2 regularization. Lipid maps show 60% lower residual lipid signal for LIPNET compared with L2 regularization. On the other hand, it can be seen that without any lipid removal, the metabolic maps are completely overwhelmed by lipid artifacts, with no visible structural details of the brain and very large quantification errors. The lipid examples of spectra show clearly more residual lipid signal by L2 regularization than by LIPNET, while in the case of no lipid removal, the metabolite spectra are heavily distorted by the large lipid signal. In addition, metabolic maps of Cr+PCr, and Glutamate, which are consistent with the previous findings, are presented in Figure S2.

**FIGURE 4:**
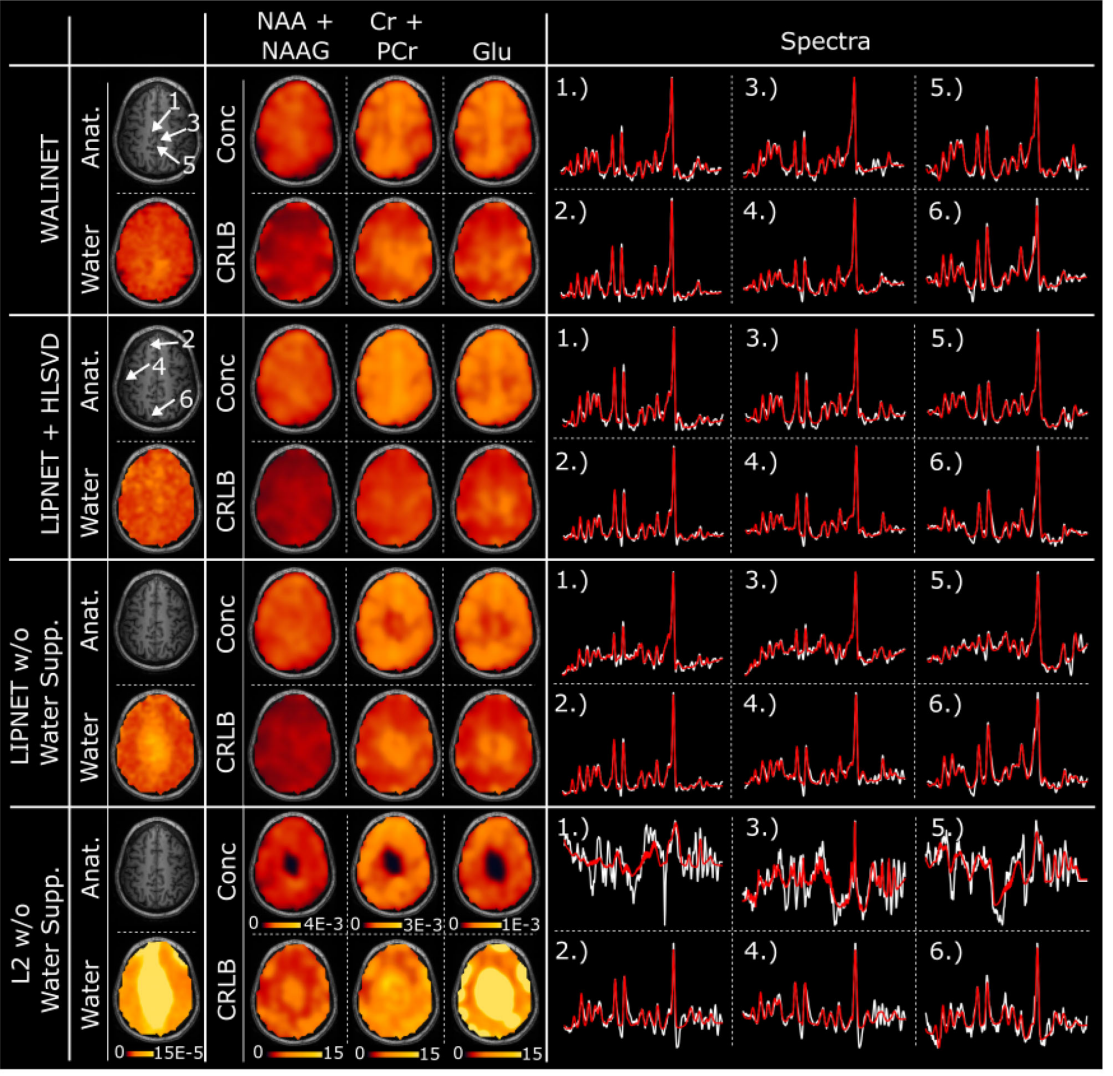
Comparison of water removal methods on in vivo 2D MRSI, including WALINET, HLSVD+LIPNET, LIPNET and L2 without water suppression. WET^11^ water suppression was used during acquisition. Metabolic maps and the corresponding CRLBs are shown for NAA+NAAG, Cr+PCr, and Glu together with maps of the residual water signal and examples of spectra (voxel locations are indicated by arrows, red line indicates LCModel^47^ fit and the white line indicates the experimental spectra).

Second, we studied the effects of different water removal methods. Figure 4 compares the water removal by WALINET and HLSVD on in vivo 2D MRSI. Similar maps of the residual water signal and metabolites are obtained for WALINET and LIPNET+HLSVD. The maps obtained without water suppression show higher residual water signal and signal dropout in the center of the brain, which is worse for L2 than LIPNET. Spectra and LCModel^47^ fit show progressively larger baseline noise and more distortion of metabolite peaks when going from WALINET to LIPNET+HLSVD, LIPNET, and L2. Examples of spectra with full spectral window and intensity range are shown in Figure S3.

Taken in combination, the results from Figures 3 and 4 indicate that WALINET and LIPNET generalize well to different acquisition schemes (2D Cartesian vs. 3D Non‐Cartesian) that were not used for the acquisition of training data.

**FIGURE 5:**
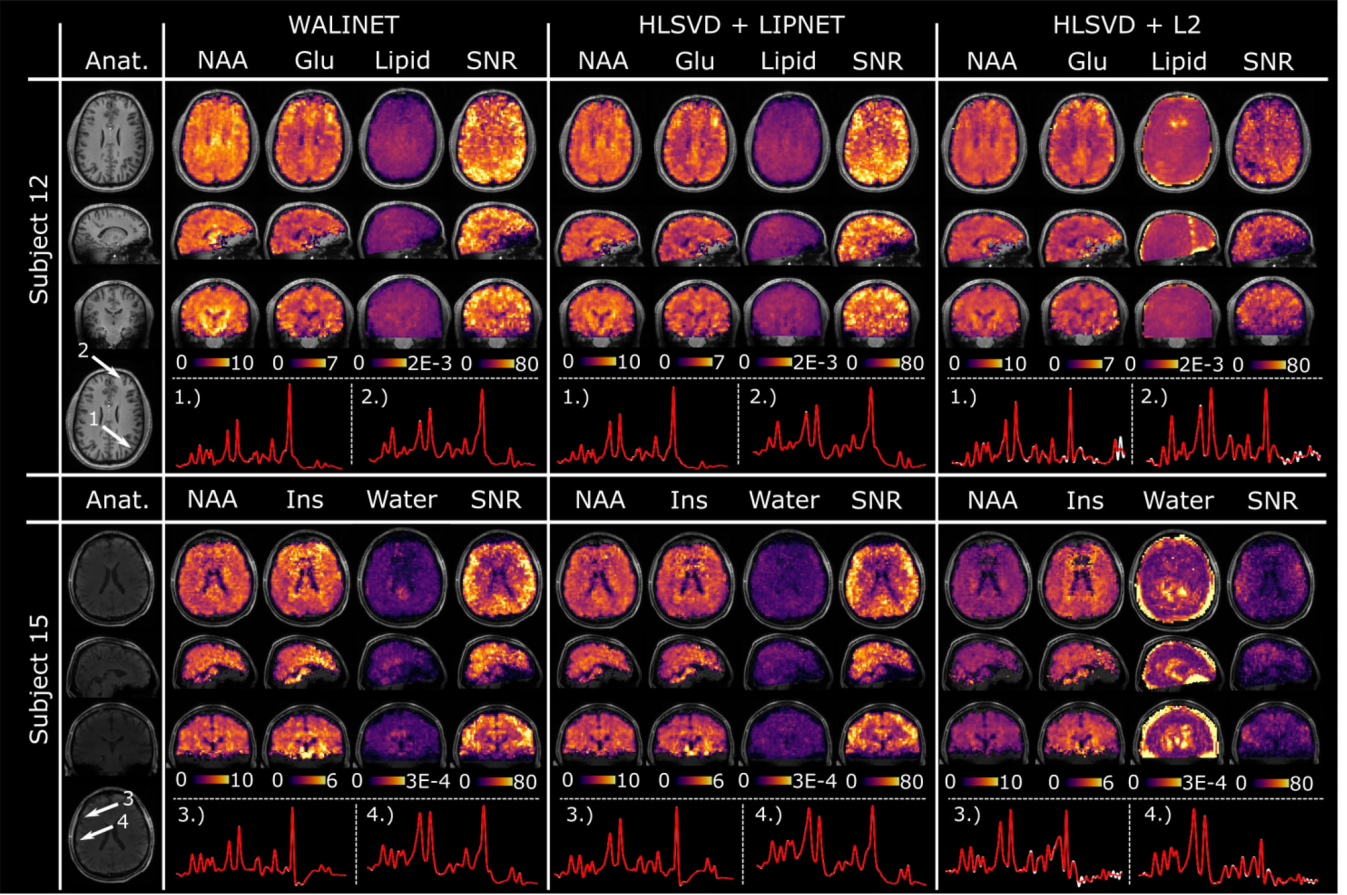
Comparison of combined water & lipid removal on in vivo 3D MRSI. Results are shown for WALINET, HLSVD+LIPNET, and HLSVD+L2, including metabolic maps for NAA, Glutamate, Inositol, residual lipid signal, residual water signal, and SNR computed by LCModel. Selected spectra from individual voxels indicated by white arrows on the anatomical images are shown at the bottom (white trace shows measured spectrum, red trace shows LCModel^47^ fit).

Third, we investigated the effects of combined water and lipid removal on 3D MRSI. Figure 5 compares the performance of WALINET, HLSVD+LIPNET, and HLSVD+L2 on in vivo 3D MRSI data from two evaluation subjects. It can be seen that WALINET and HLSVD+LIPNET provide similar metabolic maps, residual lipid & water maps, SNR, and spectra. The results provided by HLSVD+L2 show more residual lipid & water signal, lower SNR, lower gray/white matter contrast in metabolite maps. In particular, spectra obtained by HLSVD+L2 show a reduction of the NAA peak and larger residual lipid peaks.

**FIGURE 6:**
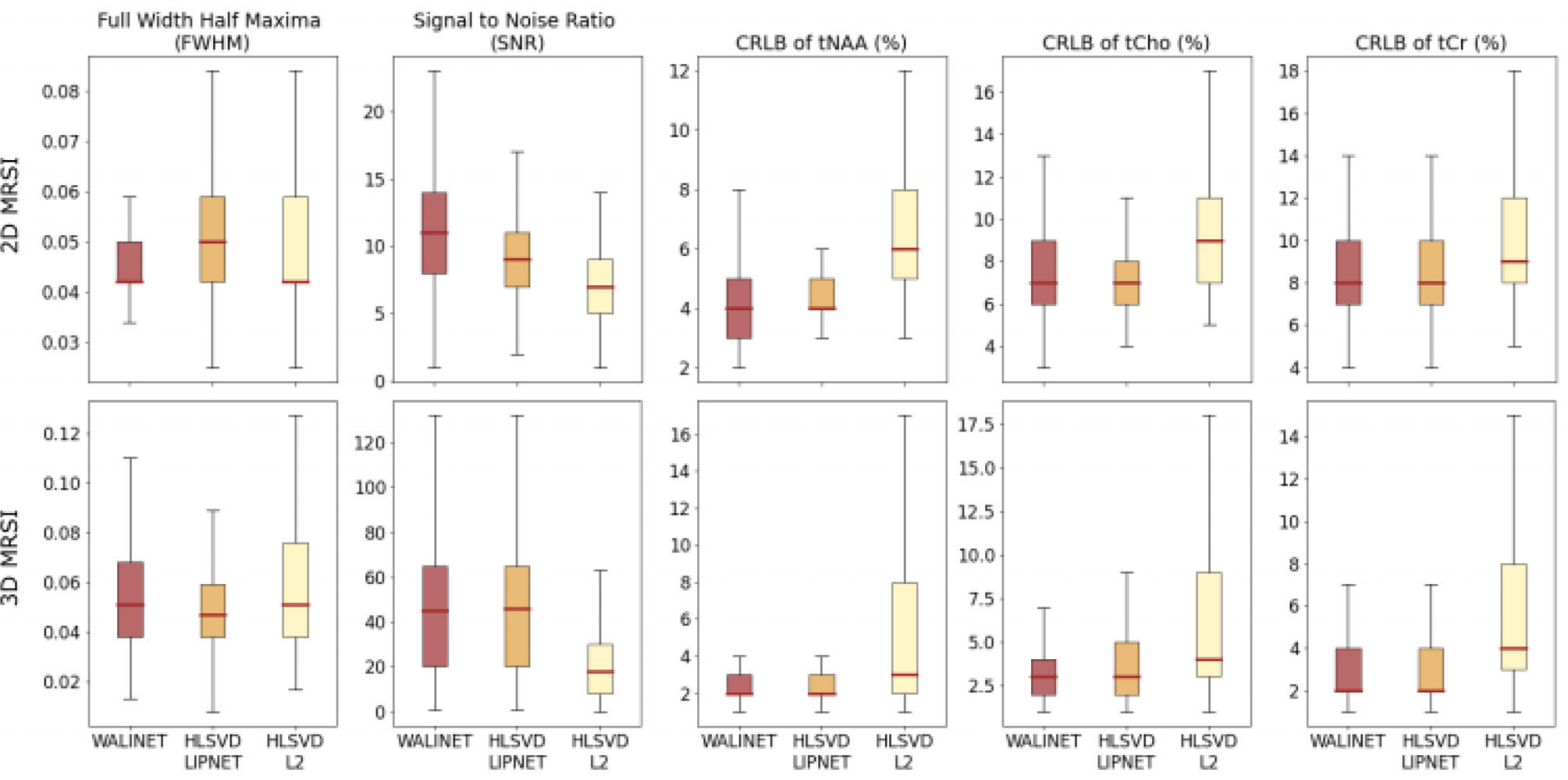
Boxplots of spectral quality metrics obtained be WALINET, HLSVD+LIPNET, and HLSVD+L2 in 2D and 3D in vivo MRSI data.

Figure 6 compares the metrics of spectral quality obtained by WALINET, HLSVD+LIPNET, and L2+HLSVD on 2D and 3D datasets. Results indicate that WALINET has similar mean SNR (11/45 in 2D/3D) and mean CRLB (NAA/Cho/Cr = 4/7/8% in 2D and 2/3/2% in 3D) compared with HLSVD+LIPNET (SNR = 9/46 in 2D/3D; CRLB of NAA/Cho/Cr = 4/7/8% in 2D and 2/3/2% in 3D). At the same time, both of these deep learning‐based methods have higher SNR and lower CRLB compared with conventional L2+HLSVD (SNR = 7/18 in 2D/3D; CRLB of NAA/Cho/Cr = 6/9/9% in 2D and 3/4/4% in 3D). Additionally, the interquartile interval and the min–max whiskers of CRLB are narrower for WALINET and HLSVD+LIPNET than HLSVD+L2. The spectral linewidths show similar mean values (0.04–0.05 ppm) for all three methods, but narrower interquartile interval and min–max whiskers for WALINET and HLSVD+LIPNET than HLSVD+L2.

The lipid removal factor and water removal factor were quantified in vivo, and simulations for each of the methods are listed in Table S1. It can be seen that WALINET and LIPNET provide in vivo a mean lipid removal factor between 25 and 45 in the brain (689–876 lipid removal factor in the scalp), and a mean water removal factor between 34 and 53.

## 4 DISCUSSION

We demonstrate WALINET, a fast and robust nuisance signal identification convolutional neural network. WALINET was trained to identify water and lipid signals in whole‐brain 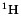
‐MRSI spectra, and simultaneously removes these signals to allow accurate quantification of metabolites. WALINET eliminates time‐consuming computations for water removal and iterative single‐subject hyperparameter optimization for lipid suppression by conventional methods. Thereby, WALINET can streamline and automate MRSI data processing for user‐friendly clinical applications.

Considering that the evolution of MRSI is toward high‐spatial resolution with large matrix size, the development of computationally efficient processing pipelines is required to keep up with the computational demands posed by the need to process increasing data size. Our evaluation showed that WALINET is considerably faster (8 s) on high‐resolution MRSI compared with conventional methods (42 min).

In addition to faster processing times, WALINET showed superior performance with more lipid removal and preserving more metabolite signal compared with state‐of‐the‐art conventional lipid removal methods. WALINET provided significantly higher data quality, effectively doubling the SNR and lowering by half quantification errors of metabolites, compared with conventional methods. WALINET and LIPNET have a lipid removal factor that is 11.19 times larger than L2, and a water removal factor that is 2.86 times larger compared with HLSVD. Compared with other methods listed in the recent consensus paper,^7^ WALINET and LIPNET provide lipid suppression factors similar to the crusher coil and ECLIPSE but without the need for special hardware. A potential caveat of WALINET and LIPINET is the lactate doublet at 1.3 ppm may be removed together with the lipid signal, hence the ability to obtain lactate maps is dependent on the lactate quadruplet at 4.1 ppm. By comparison, the nuisance signal removal using the union‐of‐space model^23^ has been shown to retain the lactate peak at 1.3 ppm.

Convolutional neural networks act as nonlinear functions that may model better lipid contamination in MRSI, while conventional methods^23^, ^29^ that assume a linear orthogonal relationship between lipids and metabolites may inadvertently remove metabolite signal when this assumption is not met. The effects of improved metabolite quantification translate into metabolite images that have better structural details. We believe that a robust self‐contained efficient nuisance signal removal method that is implemented as an independent processing step is very useful and can be combined with any MRSI processing pipeline. This may offer greater flexibility compared with methods that are fully embedded with the reconstruction of k‐space MRSI data.

While LIPNET and WALINET were exclusively trained on 3D MRSI spectra, the presented results demonstrate robust performance also on 2D MRSI test data, which differs in FID length and spectral bandwidth (echo spacing). In our experience, machine learning algorithms can extrapolate to a certain extent to out‐of‐distribution data. However, the extension of WALINET to different sequences, spectral bandwidths, and FID lengths remains future work.

At the moment, the demonstration of WALINET performance was limited to 7T ultrahigh‐field MRSI, which is the highest field approved for clinical use. Methods such as WALINET are highly relevant at 7T because the high SAR and nonuniform $B_{0}$ and $B_{1}+$ fields make pulse sequence‐based suppression of water and lipids highly impractical for whole‐brain MRSI. However, we expect that the approach and the same network can be employed with additional training at lower (3T) or higher fields. We also expect that the processing time of WALINET will be similar for higher spatial resolution of MRSI, while the time for conventional methods will linearly scale with the data size. Furthermore, removal of lipid signal is extremely important for accelerated undersampled MRSI acquisitions^48^, ^49^ where aliased lipid signal can overwhelm parallel imaging and compressed sense reconstructions. Based on its performance, WALINET has great potential for being used in combination with undersampled MRSI, and we will explore this in future work. This is suggested by the generalization of WALINET from 3D non‐Cartesian sampling to 2D Cartesian sampling that we demonstrated in this paper. In addition, we showed that the same model can be used for lipid‐only removal (LIPNET), which can be further combined with other processing methods. We also noticed that LIPNET provides some suppression of the water tail in the metabolite spectral range. This happens because there are lipid signals (at 5.1 and 4.4 ppm) close to the water peak which the network learns to remove. However, in the presence of a large residual water peak, LIPNET is not sufficient and a water‐trained network is necessary such as WALINET.

## 5 CONCLUSION

Efficient removal of water and lipid signals is key for proton MRSI‐based quantitative metabolic imaging. Convolutional neural networks such as WALINET provide an effective approach toward this goal with superior performance compared with conventional methods. We provide WALINET, including the computation of the L2 lipid operator, as a self‐contained package that can be used as a plug‐in with any MRSI processing pipeline. Because WALINET does not need specialized hardware, it can be easily disseminated to automate the MRSI processing pipeline for high‐throughput workflow in clinical applications. We anticipate that these aspects will lead to larger adoption and impact of MRSI for clinical applications and research.

## Supporting information




**Data S1.** Supporting Information.

## Data Availability

Testing data can be obtained from the authors based on reasonable request and institutional approved data sharing agreement. The code for WALINET is publicly available at http://www.github.com/weiserjpaul/WALINET.
