# Supplementary material for: WALINET: A water and lipid identification convolutional neural network for nuisance signal removal in MR spectroscopic imaging
Source: Magn Reson Med. 2024 Dec 31;93(4):1430–42. doi: 10.1002/mrm.30402 (PMC11782715; doi:10.1002/mrm.30402)
Supplement: Supplementary file 1 — Data S1. Supporting Information. [file MRM-93-1430-s001.pdf]

Supplementary Material

5 Supplementary Methods

Data Acquisition:

2D 1H-FID Cartesian MRSI data were acquired with unaccelerated elliptical phase-encoded. 3D 1H-FID-ECCENTRIC[46] was acquired with randomly positioned circular trajectories with the radius set to kmax/8 without temporal interleaving and full sampling (AF=1) of a spherical 3D k-space.

For both sequences the excitation was performed with a Shinnar-LeRoux optimized pulse[45,46] having 6.5kHz bandwidth, 1ms duration and 27° excitation flip-angle. No lipid suppression was employed in the sequence, while water suppression was achieved by a four-pulses WET method[45,46].

In addition, low-resolution water-unsuppressed MRSI were acquired as calibration scan using the same sequences but omitting WET and with a smaller matrix (22x22x11 for 3D and 22x22 for 2D) in 1:16 min:s for 3D ECCENTRIC and 2:12 min:s for 2D Cartesian. The water-unsuppressed MRSI were used for coil combination and B_0 field inhomogeneity correction.

Training Data:

First, spectra for 25 common ^{1}H metabolites were simulated using a physical model for the coupled spin systems[42,43]. An extensive dataset of 1.9×10^6 metabolite spectra was simulated for a wide range of concentrations, linewidths, noise levels, and baselines. The metabolite concentrations were distributed according to a normal distribution with a standard deviation five times greater than the mean of 1 (arbitrary units), with truncation to zero to ensure positive values. The simulation included also variations in frequency offset (-150 to 150 Hz), Voigt linewidth (4 to 50 Hz), and signal-to-noise ratio (SNR from 1 to 10). Random baselines were introduced, characterized by 10 broad gaussian components.

Second, we extracted a collection of lipid spectra from voxels within the scalp region obtained from *in-vivo* MRSI datasets. Representative head/brain/scalp masks and B0 field maps are presented in Supplementary Figure S1 . The amplitude of lipid signal from the lipid mask was varied with a scaling factor, spanning a broad interval from 10^{-2} to 10^3. In addition, the phase of the lipid signal was varied between $-\pi$ and $+\pi$. The variation of the amplitude and phase of the lipid signal mimics the sinc ringing of the point spread function. The large range of amplitude and phase variation effectively augments the *in-vivo* lipid contamination, which makes the network robust to different k-space sampling strategies. Hence, we do not need to retrain the network when changing the acquisition. This was verified by testing WALINET on MRSI data acquired with ECCENTRIC as well as cartesian phase encoding.

Third, the water signal was extracted using Hankel-Lanczos singular value decomposition (HLSVD) with a rank of 64 from voxels within the brain from the same *in-vivo* MRSI datasets. To further augment the *in-vivo* water distribution the 10 water signal components estimated by HLSVD in each voxel were randomly weighted with a factor ranging from 10^{-1} to 10^2.

To create the final training input spectra (x_1) for WALINET the simulated metabolite spectra (m) and experimentally-derived lipid & water spectra (x_2) were randomly combined. Note, that in the case of LIPNET the input training spectra combined simulated metabolite and experimentally-derived lipid spectra, without the water signal.

Water and lipid signals were extracted from 19 subjects, including 2 glioma patients. The MRSI data were acquired with the 3D ^{1}H-FID-ECCENTRIC sequence described in the Methods. 10^5 lipid & water spectra were extracted from each subject, resulting in 1.9×10^6 total metabolite + lipid & water spectra used for training. Additionally, a validation dataset included 4 other subjects.

Processing Pipeline:

For 3D ECCENTRIC the k-space sampling density was compensated based on Voronoi diagrams[50] where each k-space point is normalized by the area of its assigned Voronoi vertex. Upon the weighting of the k-space data, an inverse non-uniform discrete Fourier transform (iNUFT) was applied for each stack (kx-ky plane) within the k-space domain. Subsequently, an additional inverse fast Fourier transform (iFFT) was performed along the kz dimension to finalize the reconstruction of the MRSI data. In contrast, for 2D ^1H-FID-MRSI data a iFFT is applied on the 2D k-space datasets. For both 2D and 3D MRSI a Hamming filter was applied in k-space prior to the Fourier transform. After the transformation from k space to image space, the spectra are obtained by Fourier transform of the time dimension. Coil combination was performed with ESPIRIT[51] using sensitivity profiles computed from water un-suppressed MRSI. A correction for B_0 field inhomogeneity (Supp. Fig. S1)

For Peer Review

is computed from water un-suppressed data and applied to the coil-combined image space MRSI data, which was followed by water and lipid removal.

LCModel used a basis set simulated by NMR quantum mechanics in GAMMA[52] for twenty-two metabolites: phosphorylcholine (PCh), glycerophosphorylcholine (GPC), creatine (Cr), phosphocreatine (PCr), gamma-aminobutyric acid (GABA), glutamate (Glu), glutamine (Gln), glycine (Gly), glutathione (GSH), myo-inositol (Ins), N-acetylaspartate (NAA), N-acetyl aspartylglutamate (NAAG), scylloinositol (Sci), lactate (Lac), threonine (Thr), beta-glucose (bGlu), alanine (Ala), aspartate (Asp), ascorbate (Asc), serine (Ser), taurine (Tau), and 2-hydroxyglutarate (2HG) and a measured macromolecular background[53]. Note that during training WALINET and LIPNET learn to remove macromolecule signal, since this is present together with lipid signal in the scalp spectra used to generate training data. This was verified experimentally, as macromolecular fitting by LCModel was very close to 0 throughout the brain. The spectral fitting was done for the 1ppm-4.2ppm spectral range and the results for each voxel were used to generate metabolic images. The unsupressed water reference signal was used as quantification reference for metabolites concentrations (institutional units, I.U.) to compare metabolite levels across subjects and scanners. To assess the quality of the MRSI data and fit, linewidth (FWHM), signal-to-noise ratio (SNR), and Cramer-Rao lower bounds (CRLB) goodness of fit maps were generated.

The lipid removal factor was calculated in test subjects and in simulations. For this we integrated the absolute value of the lipid signal before and after removal: a) in the range 0.8-1.8 ppm to exclude the metabolite region (1.8-4.2 ppm) for the in-vivo data, and b) over the entire spectral range of interest 0.8-4.2 ppm in simulations, since in simulations we have the metabolite-free lipid signal over the entire range. The water removal factor was calculated by integrating the absolute water signal between 4.2-5.2 ppm, before and after the removal.

For Peer Review

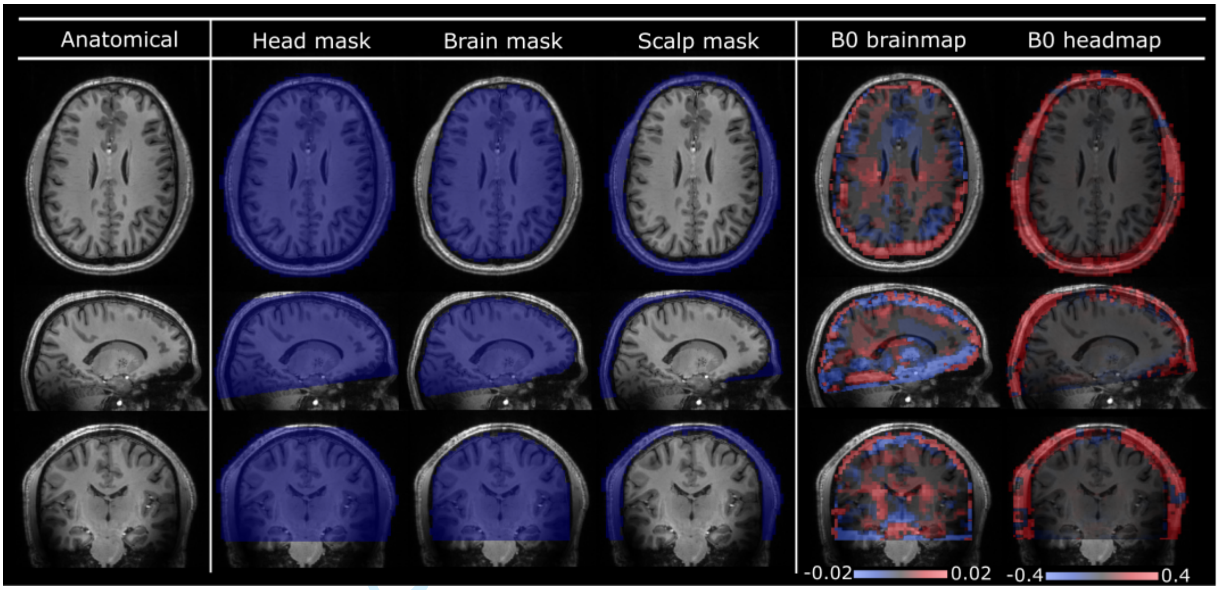

Supporting Figure S1 Examples of head/brain/scalp binary masks and the corresponding B0 field maps (specified in ppm).

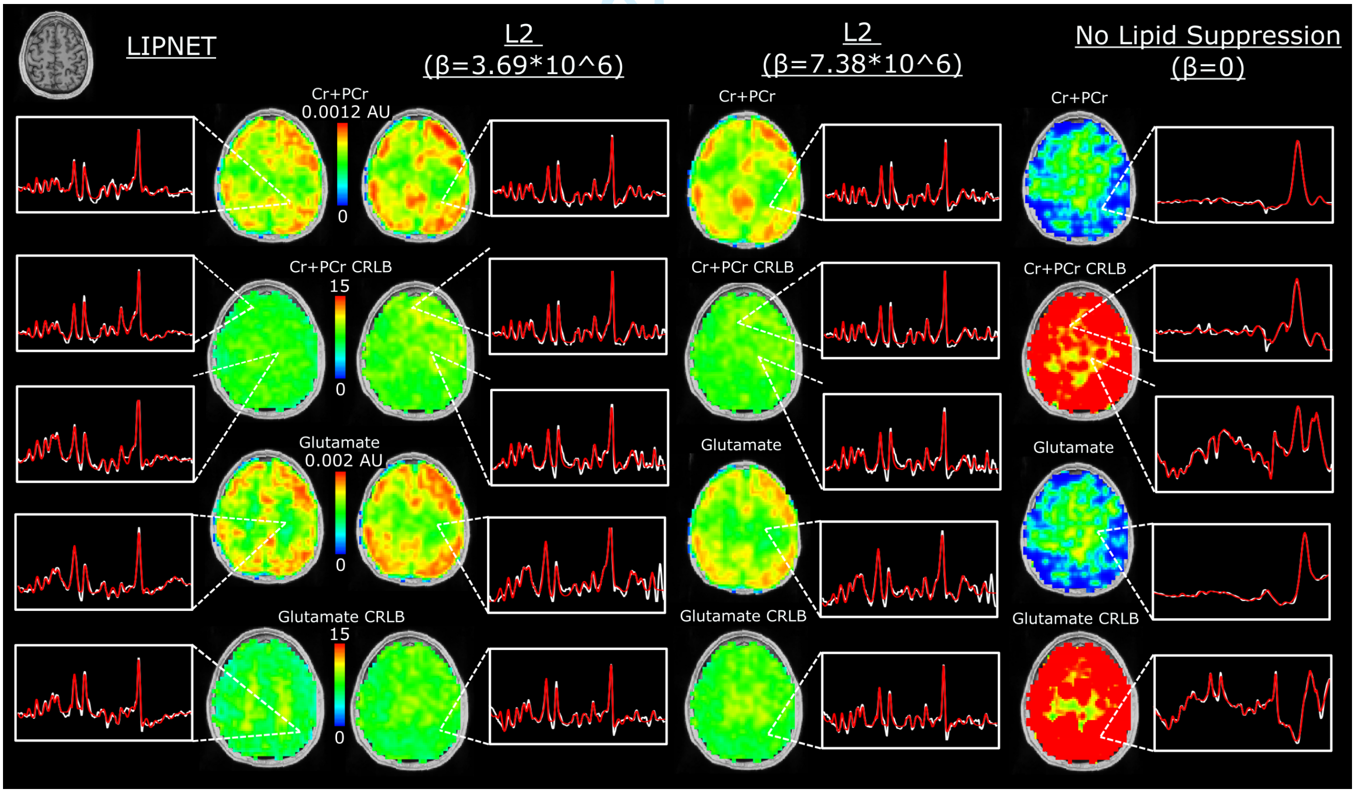

Supporting Figure S2 Comparison of lipid removal on *in-vivo* 2D MRSI by LIPNET and L2 for three values of the regularization parameter β, the optimal value ($3.69 * 10^6$), double the optimal value, and zero for no lipid suppression. Maps are shown for two metabolites Cr+PCr, Glu and their corresponding CLRB. Spectra from several brain voxels are shown for each method, the white trace shows the measured spectrum, the red trace shows LCModel fit.

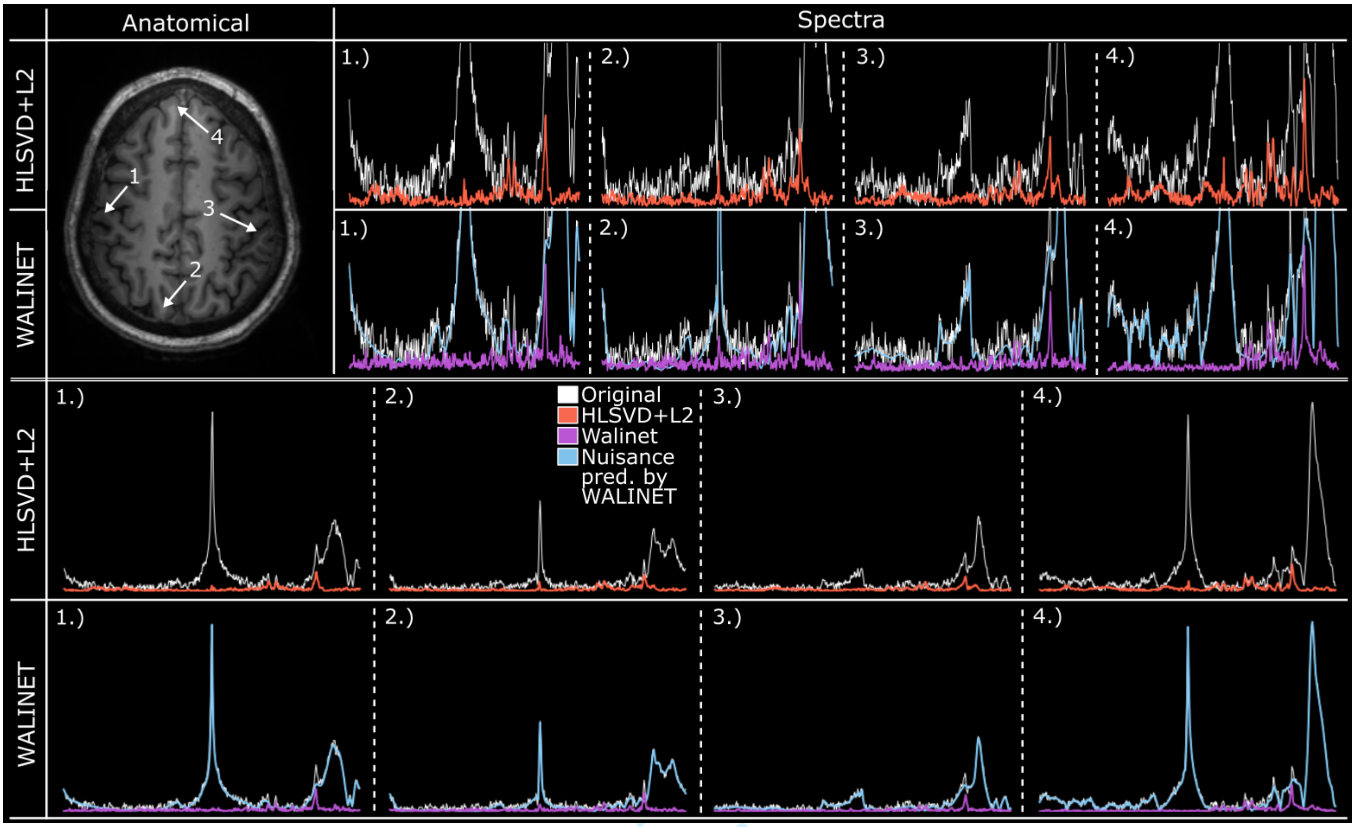

Supporting Figure S3 Comparison of water and lipid removal to WALINET on full range spectra. Each specrtum is shown enlarged on top and in full size below. The nuisance contaminated spectra is displayed in white, HLSVD+L2 processed spectra in orange, and WALINET nuisance removed spectra in purple. The from WALINET predicted nuisance signal is overlayed in light blue color.

		vol12	vol15	Simulated
**Lipid Removal Factor:** Brain mask	Mean	25.43	45.28	235.55
		20.72	25.61	17.68
	Median	20.57	35.60	118.51
		18.07	22.41	8.97
**Lipid Removal Factor:** Head mask	Mean	689.23	876.14	235.55
		76.81	73.59	17.68
	Median	78.69	156.73	118.51
		33.56	37.36	8.97
**Water Removal Factor:** Brain mask	Mean	34.18	53.09	224.20
		18.84	18.53	91.51
	Median	17.19	26.40	69.81
		15.54	16.43	12.54

Supporting Table S1 Lipid and Water Removal Factor Comparison. WALINET results are shown in red, HLSVD+L2 in yellow. Lipid removal factors were computed in the head and brain, water removal factor was in the brain only. Results are shown for to subjects and on simulated data.
